# Family history of venous thromboembolism is a risk factor for venous thromboembolism in combined oral contraceptive users: a nationwide case-control study

**DOI:** 10.1186/s12959-015-0065-x

**Published:** 2015-10-21

**Authors:** Bengt Zöller, Henrik Ohlsson, Jan Sundquist, Kristina Sundquist

**Affiliations:** Center for Primary Health Care Research, Lund University/Region Skåne, Skåne University Hospital, CRC, Building 28, Floor 11, Jan Waldenströms gata 35, S-205 02 Malmö, Sweden; Stanford Prevention Research Center, Stanford University School of Medicine, Palo Alto, CA USA

**Keywords:** Oral contraceptives, Venous thromboembolism, Epidemiology, Genetics

## Abstract

**Background:**

The aim was to assess the risk of venous thromboembolism (VTE) associated with use of combined oral contraceptives (COCs) in women with a family history of VTE.

**Methods:**

The study is a Swedish nationwide case-control study based on the Multigeneration register, the Swedish Hospital Discharge Register, the Outpatient Care Register, and the Swedish Prescribed Drug Register. Cases (n = 2,311) were non-pregnant Swedish women aged 15-49 with first VTE diagnoses between January 2006 and December 2010. Five controls without VTE were matched to each case on age and education level. Conditional logistic regression examined the associations with VTE with determination of odds ratio (OR) for first VTE diagnosis. Effect modification was assessed by interaction testing.

**Results:**

Both among controls (14.6 % vs. 4.5 %; p < 0.0001) and cases (27.2 % vs. 8.8 %; p < 0.0001) COC use was more common in women without a family history of VTE compared with women with a family history of VTE. In a multivariate conditional logistic regression model the OR for VTE was 2.53 (95 % CI 2.23-2.87) for COC users and 2.38 (2.09-2.71) for individuals with a family history of VTE. The OR for VTE for COC users with a family history of VTE was 6.02 (5.02-7.22). There was no significant interaction between family history of VTE and COC use (OR 0.92, 0.57-1.46).

**Conclusions:**

Family history of VTE is a risk factor for VTE in women using COCs. The low prevalence of COC use among women with a family history of VTE suggests that family history of VTE is considered when COCs are prescribed in Sweden. The present study may therefore even underestimate the importance of family history of VTE. The lack of interaction indicates that the risk of COC use in women with family history of VTE is determined by the product of the ORs for family history and COC use.

**Electronic supplementary material:**

The online version of this article (doi:10.1186/s12959-015-0065-x) contains supplementary material, which is available to authorized users.

## Introduction

The first report of an increased risk of venous thrombosis associated with oral contraceptives appeared in 1961 [1]. Since then, several large studies have confirmed a two-fold to six-fold increased risk of deep venous thrombosis associated with current oral contraceptive use [2–15]. The thrombotic risk of oral contraceptives is dependent on the oestrogen dose and type of progestogen [16]. The risk of venous thromboembolism (VTE) in women using combined oral contraceptives (COCs) is attributed to changes in haemostasis [17]. These changes may have a greater impact in women with inherited thrombophilic defects (antithrombin deficiency, protein C deficiency, protein S deficiency, factor V Leiden) [18–26]. While the absolute risks for VTE among healthy women using COC are low, their use by women with inherited thrombophilic defects can pose significantly increased health risks. Recognizing this increased VTE risk, WHO recommends against use of COCs among women with these conditions. However, because of the low prevalence of thrombophilic defects and the high cost of screening, routine assessments for these conditions are not endorsed by WHO [27]. Several reports have investigated whether family history of VTE could be used to identify women with thrombophilic defects, but the sensitivity and specificity of finding thrombophilic defects are too low [28, 29]. In fact, Bezemar found that only 30 % of patients with a family history of VTE have an identified thrombophilic defect [30].

Several studies have shown that family history of VTE is a common and strong risk factor for VTE even in the absence of known thrombophilic defects [30–33]. Moreover, family history of VTE is more strongly related to shared biological (genetic) factors than to environmental factors [34, 35]. In a study of 50 women with VTE using oral contraceptives, 16 % had a family history of VTE [36]. In a case-control study of women with VTE aged 18-64 years, self-reported family history of VTE in combination with COC use gave a very high odds ratio of (OR) for VTE of 15.3 (95 % confidence interval [CI] = 6.1-38), compared with non-users without a family history of VTE [37]. Potential drawbacks of this case-control study were the possibility of recall bias, the inclusion of older women who do not use COCs, and the failure to exclude women who had been sterilized, or hysterectomized. No large nationwide family study has determined whether the importance of family history of VTE is a risk factor for VTE in patients treated with COCs.

We assessed the risk of VTE in users of oral contraceptives with a family history of VTE in Sweden using nationwide registers. The aim was to determine the importance of family history of VTE as a predictor for VTE in women using COCs.

## Material and methods

### Details of ethics approval

The study was approved by the Ethics Committee of Lund University, Sweden (approval number 409/2008, with amendments approved on September 1 2009 and January 22 2010). It was performed in compliance with the Declaration of Helsinki. Consent was not obtained but the presented data are anonymised and there is no risk of identification.

### Settings

We linked comprehensive register and health care data from multiple nationwide Swedish sources to form a database using the unique individual Swedish 10-digit personal ID numbers assigned to all residents. These ID numbers were replaced with serial numbers in order to preserve confidentiality. Our database contained the following sources [38–42]: the Swedish Hospital Discharge Register, which included all hospitalizations in Sweden in 1964-2010; the Multi-Generation Register, which included information on family relationships for all individuals born in Sweden in 1932 and later; the Swedish Prescribed Drug Register, which included all prescriptions in Sweden picked up by patients between July 1 2005 and December 31 2010; the Swedish Cause of Death Register, which contained all causes of death and times of death from 1961-2010; and the Outpatient Care Register, which included information from outpatient clinics covering all geographic regions in Sweden from 2001-2010.

### Inclusion and exclusion criteria

The dataset for these analyses was created by identifying from the Swedish Hospital Discharge Register and the Outpatient Care Register all females aged 15-49 with diagnoses of VTE during the period 2006 to 2010. We defined VTE based on the following ICD-10 codes: I636 (cerebral infarction due to cerebral vein thrombosis), I676 (cerebral vein thrombosis), I80 (venous thrombosis of the lower extremities [except I80.0, i.e. superficial thrombophlebitis]), I81 (portal vein thrombosis), I82 (other venous embolism or thrombosis) and I26 (pulmonary embolism). We used only main diagnoses to guarantee high validity (*N* = 7,795 VTE cases). Numbers of cases by each kind of venous thromboembolism are presented in Table 1. Furthermore we required that the patients retrieved from the pharmacy an antithrombotic agent within 14 days from the date of their VTE diagnosis (requirement 1). Antithrombotic agents were defined according to the following ATC codes: B01AA, B01AB (except B01AB02), B01AE, B01AF and B01AX. We also required that both the father and mother were registered in the Multi-Generation Register (requirement 2). Individuals with previously registered diagnoses of any type of VTE in the Swedish Hospital Discharge Register (from 1964) and/or the Outpatient Care Register (from 2001) were excluded (requirement 3). Any type of VTE was defined by the ICD-7, ICD-8, ICD-9 and ICD-10 codes found in Additional file 1: Table S1 [32–35]. Individuals registered in the Swedish Hospital Discharge Register with the following prior to their VTE diagnosis were also excluded (requirement 4): Hysterectomy (surgical procedure codes LCD, 7220, 7221, 7222 and 7223), Bilateral oophorectomy (surgical procedure codes LAE20, LAE21, LAF10, LAF11, 7021, 7022, 7031 and 7032), Unilateral oophorectomy twice (surgical procedure codes LAF00, LAF01, 7020 and 7030), sterilization (surgical procedure codes LGA00-LGA98, 7150, 7151, and 7152), coronary heart disease (ICD10: I20-I25; ICD9: 410-414; and ICD8: 410-414), Heart failure (ICD10: I50, ICD9: 428, ICD8: 428), or cerebrovascular disease (ICD10: I60-I69; ICD9: 430-438; and ICD8: 430-438). The following exclusion criteria were additionally applied: diagnosis of any form of cancer in the Swedish Hospital Discharge Register within 5 years before or 1 year after their VTE diagnosis (cancer defined by the following ICD 10 codes: C00-C99) (requirement 5); treatment for infertility (ATC code G03G in the Swedish Prescribed Drug Register) (requirement 6); registered pregnancy within 9 months before or 3 months after the date of the VTE diagnosis (requirement 7); women who stopped use of any contraceptives prior to the VTE diagnosis according to the daily defined dose (DDD) from last retrieved recipe (requirement 8). We defined contraceptives by the following ATC codes: G03AA (except G03AA13) and G03AB. From the date the individual retrieved the drug from the pharmacy we used the DDD in order to calculate when they stopped using the drug. For example, if an individual retrieved the drug on January 1 2006 and the number of DDDs were 90 we considered the individual to have stopped using the drug 90 days after January 1 2006. However, if an individual retrieved a new COC within the 90 day period or less than 30 days after the 90 day period we continued to consider the individual as a user of contraceptives until the next period of use ended. Individuals who stopped using contraceptives prior to their VTE were excluded from the study. As the Prescribed Drug Register only contains data from July 1 2005 onwards we used a 6 month wash-out period. Hence, a VTE case could only be defined from the January 1 2006. In total we included 2,409 cases with VTE. In total, 5386 cases with VTE were excluded due to the mentioned exclusion criteria. Numbers of cases and controls excluded by each requirement (1-8) are shown in Table 2.

**Table 1:** Numbers of cases by each kinds of venous thromboembolism

ICD-10	Before exclusions (N = 7,795)	After exclusions (N = 2,311)
I676	119 (2 %)	27 (1 %)
I80	4,545 (58 %)	1,156 (50 %)
I81	76 (1 %)	20 (1 %)
I82	1,143 (15 %)	358 (15 %)
I26	1,887 (24 %)	746 (32 %)

**Table 2:** Numbers of cases and controls excluded by each requirement (1-8)

	Cases	Controls
Start	7,795	2,495,994
(1) Antithrombotic agent within 14 days	2,964	-
(2) Father and mother were not registered in the Multi-Generation Register	971	404,747
(3) Individuals with previously registered diagnoses of any type of VTE	1,560	15,334
(4) Hysterectomy	240	17,362
(4) Bilateral oophorectomy/Unilateral oophorectomy twice	75	3,817
(4) Sterilization	242	29.331
(4) Coronary heart disease/Heart failure/Cerebrovascular disease	191	6,915
(5) Cancer	373	8,957
(6) Infertility	240	37,239
(7) Pregnancy	343	56,043
(8) Women who stopped use of any contraceptives prior to the VTE diagnosis	982	312,099
Unable to match with 5 controls	98	-
Included in the analysis	2,311	11,555

Note that excluded and included patients will not sum up to 7,795 as VTE-cases could be excluded due to several exclusion criteria

Each case was matched to five controls based on year of birth and education level. Socioeconomic data on education correlate with lifestyle factors [39]. Adjusting for education could help to diminish confounding by lifestyle factors [39]. Education level was categorized into three groups: low (0-9 years), middle (10-11 years) and high (12 years or more). For individuals 25 years and younger we selected the highest education level achieved by either the mother or father. In order to be eligible to be a control individual we applied the same criteria as for cases (requirements 2-8). Furthermore, the control individual had to be alive and registered in Sweden at the time of the case’s VTE diagnosis. An individual could only be selected as a control individual once. Moreover, 98 cases could not be matched to five controls and were excluded from the analysis. In total we included 2,311 cases and 11,555 controls in the analyses (Table 2).

### Statistical analysis

As the main predictor variables in the models we used contraceptive use and family history of VTE. Contraceptive use was defined as described above; however, in order to be defined as a user cases had to be using contraceptives at the time of their VTE diagnosis. Controls had to be using contraceptives on the day of the VTE diagnosis in the corresponding case. Family history was defined as any type of VTE (see definition above) among first-degree relatives (mother, father and/or sibling(s)). The VTE in relatives had to be registered prior to the contraceptive prescription date for the case/control and in situations where the individual had no contraceptive use prior to the date of the VTE diagnosis in cases.

We used conditional logistic regression in order to study the effects of contraceptives, family history of VTE and their interaction on VTE. In models A1 and A2 we separately investigated the effects of contraceptives and family history of VTE on VTE, respectively. In model B we included both variables in the same model. Model C also included the interaction between contraceptives and family history of VTE. As the results from the conditional logistic regression are on the multiplicative scale we also used a hierarchical regression model (i.e., using the identity link) in order to get the interaction results on an additive scale.

In additional analyses, using conditional logistic regression, we investigated whether different generations of combined oral contraceptives had different effects. Both fixed combination (G03AA) and sequential preparations (G03AB) were considered. Contraceptives were categorized into five groups according progestogen content [43]: first-generation progesterone (ATC codes: G03AA03 [lynestrenol, G03AA05 [norethisterone], G03AA01 [ethynodiol], G03AB02 [lynestrenol], G03AB04 [norethisterone]); second-generation progesterone (ATC codes: G03AA06 [norgestrel], G03AA07 [levonorgestrel], G03AB03 [levonorgestrel]); third-generation progesterone (G03AA09 [desogestrel], G03AA11 [norgestimate], G03AB05 [desogestrel], G03AB06 [gestodene]); fourth-generation progesterone (ATC code: G03AA12 [drospirenone]); and undefined (ATC code: G03AA14 [nomegestrol]). In the models we created one term for contraceptives (yes/no) and one categorical variable defining each generation. We used the first generation as the reference in the analysis. Furthermore, we explored whether the effect of contraceptives on VTE was stronger among individuals who were newly prescribed contraceptives. We arbitrarily use 6 months prior to the date of VTE diagnosis as the cut-off. Individuals who had used contraceptives for less than 6 months were defined as new users. Finally, we investigated whether the effect of contraceptives was different for diagnosis of VTE at different ages. We included an interaction term between contraceptives and age at VTE diagnosis (mean 36.4 years). In the models the main effect of age is not included but only the interaction between COC use and age [44]. All statistical analyses were performed in SAS 9.3.

## Results

Table 3 shows descriptive statistics for all 2,311 included female VTE patients in the Swedish population (age 15-49 years) and the 11,555 controls (matched on age and education level). The mean age of cases was 36.4 years (standard deviation 9.5). Both among controls (14.6 % vs. 4.5 %; *p* <0.0001) and cases (27.2 % vs. 8.8 %; *p* <0.0001) oral contraceptive use was more common in women without a family history of VTE compared with women with a family history of VTE.

**Table 3:** Descriptive statistics for all 2,311 females (age 15-49 years) with VTE in the Swedish population and their matched controls

	CASES			CONTROLS^a^		
	Contraceptives			Contraceptives		
	No	Yes	All	No	Yes	All
Age (years)^b^	36.4 (9.5)^a^					
No VTE in relative	1,395 (72.9)	520 (27.2)	1,915 (82.9)	9,013 (85.5)	1,535 (14.6)	10,548 (91.3)
VTE in relative	361 (91.2)	35 (8.8)	396 (17.1)	962 (95.5)	45 (4.5)	1,007 (8.7)
	1,756 (76.0)	555 (24.0)	2,311	9,975 (86.3)	1,580 (13.7)	11,555

Numbers of cases are shown with percentages in brackets. ^a^Controls were

matched on age and education. ^b^The mean age (standard deviation within brackets)

### Risk of VTE in COC users and women with a family history of VTE

Table 4 shows ORs for VTE. In model A1 COC use gave an OR of 2.40 for VTE. In model A2 family history of VTE gave an OR of 2.23 for VTE. In the combined model B, the ORs for VTE were significant for both COC use (OR = 2.53) and family history of VTE (OR = 2.38). Model C included an interaction term. However, there was no significant multiplicative interaction between COC use and family history of VTE. Table 5 shows the results from the hierarchical regression model. There was no significant additive interaction between family history of VTE and COC use. The OR for COC users with a family history of VTE was 6.02 (95 % CI 5.02; 7.22). The OR of 6.02 is the OR for COC users with a family history compared to non-COC users without a family history (Model B in Table 4 (2.53*2.38)).

**Table 4:** Results from the conditional logistic regression analysis of 2,311 females (age 15-49 years) with VTE in the Swedish population and their matched controls

	Model A1 and A2	Model B	Model C
	Odds ratio	Odds ratio	Odds ratio
Contraceptives	2.40 (2.12-2.72)^a^	2.53 (2.23-2.87)	2.54 (2.23-2.90)
VTE in relative	2.23 (1.96-2.53)^b^	2.38 (2.09-2.71)	2.39 (2.09-2.74)
VTE in relative ^*^Contraceptives			0.92 (0.57-1.46)

In models A1^a^ and A2^b^ we separately investigated the effect of contraceptives and family history of VTE, respectively, on VTE. Model B included both variables. Model C also included the interaction between contraceptives and family history of VTE.

*=times sign.

**Table 5:** Test for additive interaction between family history of VTE and COC use

	Beta (SE)
Contraceptives	0.12 (0.009)
VTE in relative	0.14 (0.011)
Interaction between VTE in relative and Contraceptives	0.05 (0.043)^a^

Results from a regression model with an identity link for 2,311 females (age 15-49 years) with VTE in the Swedish population and their matched controls

^a^There was no significant additive interaction between family history of VTE and COC use, p-value was 0.27

### Different generations of COCs

Table 6 shows the results for risk of VTE for different generations of COCs. Only generation 4 had a significant higher OR than generation 1. The estimated OR was highest for generation 4 (OR = 3.58). Additional file 2: Table S2 shows the frequency of family history of VTE for different generations of COC. The frequency of family history of VTE for users of the different generations of COCs was as follows: first generation 3.71 %, second generation 3.17 %, third generation 3.45 %, fourth generation 3.18 % and undefined generation 4.99 %. Thus, the increased OR for generation 4 was not explained by family history of VTE.

**Table 6:** VTE risk for contraceptives containing different generations of progestogens

	OR (95 % CI)	Estimated OR
Generation 1	Ref	2.00 (1.44-2.79)
Generation 2	1.04 (0.74-1.48)	2.09 (1.29-3.37)^a^
Generation 3	1.23 (0.84-1.80)	2.46 (1.48-4.09)^a^
Generation 4	1.79 (1.22-2.62)	3.58 (2.16-5.93)^a^
Undefined generation	1.64 (0.52-5.15)	3.29 (1.00-10.82)^a^

Results from conditional logistic regression for 2,311 females with VTE in the Swedish population and their matched controls

^a^Calculated by multiplying the term (i.e., 1.04, 1.23, 1.79, and 1.64, respectively) for each generation and the term that defines use of contraceptives (i.e.; 2.00)

### New users of COC

Table 7 shows the risk of VTE according to time from start of COC use. There was a significant interaction between COC use and new users of COC. This means that the risk of VTE was highest during the first 6 months of COC use (OR = 3.53), compared to an increased odds of 2.12 if you had used COC for more than 6 months.

**Table 7:** Risk of VTE according to duration of COC use

	OR (95 % CI)	Estimated OR
Contraceptives	2.12 (1.84-2.44)	
Users of COC for more than 6 months	Ref	2.12 (1.84-2.44)
New Users of COC	1.67 (1.33-2.09)^a^	3.53 (2.56-4.36)

Results from conditional logistic regression for 2,311 females (age 15-49 years) with VTE in the Swedish population and their matched controls

^a^Interaction between COC use and new users of COC

### Age and COC use

Table 8 shows the testing for interaction between COC use and age. There was no interaction between COC use and age (Table 8). Thus, the OR is independent of the age.

**Table 8:** Testing for interaction between COC use and age

	OR (95 % CI)
Contraceptives	2.40 (2.08-2.76)
Interaction between COC use with age	1.00 (0.99-1.01)

Results from conditional logistic regression for 2,311 females (age 15-49 years) with VTE in the Swedish population and their matched controls

### Sensitivity analysis

In Table 9, a sensitivity analysis including all potential cases (7,795) matched to 5 controls is presented. The results are not different to any major degree compared to the main results after exclusions presented in Table 4.

**Table 9:** Sensitivity analysis including all potential cases (7,795) matched to 5 controls

	Model A1 and A2	Model B	Model C
	Odds ratio	Odds ratio	Odds ratio
Contraceptives	1.92 (1.76-2.10)	2.00 (1.82-2.18)	1.97 (1.80-2.16)
VTE in relative	2.42 (2.24-2.60)	2.47 (2.29-2.66)	2.45 (2.27-2.64)
VTE in relative *Contraceptives			1.23 (0.84-1.82)

*=times sign

## Discussion

### Main findings

In this study we showed that family history of VTE is an independent risk factor for VTE among COC users. The effects of family history of VTE and use of COC were quite similar (OR ~2.5) (Table 4). We did not find an interaction between family history of VTE and COC use (Tables 4 and 5). The lack of interaction indicates that the risk of COC use in women with family history of VTE is determined by the product of the odds ratios for family history and COC use. This is similar as for the combination of COC use and carriership of Factor V Leiden [18, 19]. However, this could be due to the fact that among the 2,311 individuals with VTE, only 35 had both a family history of VTE and were COC users. Interestingly, the prevalence of family history of VTE was lower among COC users (both controls and cases) compared to non-COC users, suggesting that clinicians in Sweden consider family history of VTE when prescribing COCs. As Swedish clinicians do not prescribe COCs to women with a family history of VTE to a high extent, the result may be an underestimation of the importance of family history of VTE.

### Strengths and limitations

The present study has a number of strengths. These include nationwide coverage in a country of high medical standards, and diagnosis of patients by specialists during extended examinations in clinics [38–41]. Data in the Swedish registers are remarkably complete. In 2001, personal numbers were missing in only 0.4 % of hospitalisations and main diagnoses in 0.9 % of hospitalisations [38]. The Swedish Hospital Discharge Register was started in 1964, and has had nationwide coverage since 1987. Thus, the information on the exposure was complete and had been gathered for purposes other than scientific analysis, eliminating the recall bias that is common in case-control studies [39]. Importantly, the Multi-Generation Register is a validated source that has been proved to be reliable in the study of many familial diseases [39–42]. Furthermore, we eliminated the problem of left censoring by measuring use of combined oral contraceptives over a 6 month period before our study started. The Swedish Hospital Discharge Register has nearly 90 % overall validity [38–41]. The validity for cardiovascular disorders such as VTE, myocardial infarction and stroke is around 95 % [38–41, 45, 46]. The Swedish outpatient register however, has not been validated. We were able to validate venous thromboembolic events by linking individual data on diagnoses to data on subsequent anticoagulation therapy. We restricted the analysis to cases that retrieved from the pharmacy an antithrombotic agent within 14 days from the date of their VTE diagnosis, which makes diagnosis of VTE highly probable.

This study does, however, have some limitations. We could not control for body mass index and smoking. We did not have access to data for thrombophilic defects, but family history is known to be a risk factor for VTE even in the presence of thrombophilia [30]. It is also possible that we underestimated the importance of family history as the present data suggest that COCs are less often prescribed to women with a family history of VTE. Thus, our study is conservative in its estimation of the importance of family history of VTE in COC users. We used a washout period of 6 months to include only women with new prescriptions of COC. However, we do not know if the included women used COCs before the washout period.

### Interpretation

Our results are in agreement with a previous case-control study [37]. That study showed a higher risk of VTE for the combination of family history of VTE and COC use than in the present study (OR = 15.3 vs. OR = 6.02). This might be due to the fact that COC use was a stronger risk factor for VTE in that study than in the present study, possibly due to recall and selection bias in the previous study. Our findings are also in agreement with previous reports that COCs have a multiplicative impact on individuals with thrombophilic defects [18–26]. However, only 30 % of VTE cases with a family history of VTE are explained by the known major thrombophilic defects [30]. The occurrence of VTE in patients with a positive family history of VTE is likely to have an important genetic contribution [30–35]. Thus, family history may signal the presence of unknown genetic defects in the family, which may increase the risk of VTE in women using COCs.

Another result in the present study worth mentioning is our confirmation of previous findings that the OR for VTE is highest during the initial period after starting COC use, with a significant interaction between COC use and duration of COC use (Table 7) [47, 48]. The cause of this interaction remains to be determined. We also investigated whether the prothrombotic effect of COC use differed depending on age. There was no interaction between COC use and age, indicating that the prothrombotic effect of COC use on VTE risk was independent of age. However, as the absolute VTE risk increases with age, older women using COCs will have a higher absolute risk of VTE than younger women using COCs [48].

Another subject of interest is whether the VTE risk associated with COC use is dependent on progesterone content [16, 49]. In the present study only COCs containing drospirenone (i.e. fourth-generation COCs) had higher ORs than COCs containing first-generation progesterone. The present study confirms recent studies indicating an increased risk of VTE for COCs containing drospirenone [50, 51]. However, the subgroup analysis should be interpreted with caution because the present study suggests that clinicians take VTE risk factors such as family history of VTE into account when prescribing COCs. We found no significant difference between COCs containing first- and second-generation progestogens or between COCs containing first- and third-generation progestogens. Several previous reports found a higher risk of VTE for COCs containing third- compared to second-generation progestogens [2–4, 9, 12, 19], and clinicians might therefore be less prone to prescribe COCs containing third-generation progestogens to women whom they think have an increased risk of VTE. Farmer et al. found no increased risk of VTE for third-generation compared to second-generation oral contraceptives [5]. Shapiro and Dinger suggest that the increased risk of VTE in COC users is a class effect independent of the progestogen used [52]. The third-generation progestogen norgestimate is difficult to classify as it is partly converted to levonorgestrel in the human body [53]. It is therefore sometimes classified as a second-generation progestogen. In the present study we included norgestimate among the third-generation progestogens. The increased VTE risk for fourth-generation COCs was not due to an increased prevalence of family history of VTE in users of fourth-generation COCs (Additional file 2: Table S2).

## Conclusion

Family history of VTE is a risk factor for VTE in women using COCs. The lower prevalence of COC use among women with a family history of VTE suggests that family history of VTE is considered to some degree when COCs are prescribed in Sweden. The lack of interaction indicates that the risk of COC use in women with family history of VTE is determined by the product of the odds ratios for family history and COC use. Although the present study was limited to Sweden, the Swedish population is genetically closely related to German and British people, and the results from Swedish nationwide family studies are likely to be valid for many persons of white origin in Europe and the United States [39].

## Additional files

Additional file 1: Table S1. ICD-7, ICD-8, ICD-9 and ICD-10 codes used to define family history of VTE, i.e. VTE in sibling and/or parent. (DOCX 61 kb) Additional file 2: Table S2. Family history of VTE in relation to different generations of COC. (DOCX 51 kb)

## Abbreviations

VTE: Venous thromboembolism
OR: Odds ratio
CI: Confidence interval
COCs: Combined oral contraceptives
ICD: International Classification of Diseases
