# Supplementary material for: Family history of venous thromboembolism is a risk factor for venous thromboembolism in combined oral contraceptive users: a nationwide case-control study
Source: Thromb J. 2015 Oct 21;13:34. doi: 10.1186/s12959-015-0065-x (PMC4617955; doi:10.1186/s12959-015-0065-x)
Supplement: Additional file 1: Table S1. — ICD-7, ICD-8, ICD-9 and ICD-10 codes used to define family history of VTE, i.e. VTE in sibling and/or parent. (DOCX 61 kb) [file 12959_2015_65_MOESM1_ESM.docx]

Supplementary Table 1. ICD-7, ICD-8, ICD-9 and ICD-10 codes used to define family history of VTE , i.e. VTE in sibling and/or parent.

|  | ICD-7 | ICD-8 | ICD-9 | ICD-10 |
| --- | --- | --- | --- | --- |
| Pulmonary embolism | 465 | 450 | 415B, 416W | I26 |
| Venous thrombosis of the lower extremities | 463 | 451 | 451 | I80 |
| Portal vein thrombosis | 583.00 | 452 | 452 | I81 |
| Cerebral vein thrombosis | 334,40, 334,50 | 321 | 437G | I63.6, I676 |
| Other venous embolism or thrombosis | 464, 466 | 453 | 453 | I82 |
| Pregnancy and postpartum related venous thromboembolism | 682, 684 | 671, 673.9 | 671C, 671D, 671E, 671F, 671X, 673C | O222, O223, O225, O229, O870, O871, O873, O879, O882 |
| Abortion related thrombosis |  |  | 639G | O082. O087 |
