# Supplementary material for: Family history of venous thromboembolism is a risk factor for venous thromboembolism in combined oral contraceptive users: a nationwide case-control study
Source: Thromb J. 2015 Oct 21;13:34. doi: 10.1186/s12959-015-0065-x (PMC4617955; doi:10.1186/s12959-015-0065-x)
Supplement: Additional file 2: Table S2. — Family history of VTE in relation to different generations of COC. (DOCX 51 kb) [file 12959_2015_65_MOESM2_ESM.docx]

Supplementary Table 2. Family history of VTE in relation to different generations of COC.

|  |  |  |  |  |  |  |  |
| --- | --- | --- | --- | --- | --- | --- | --- |
|  |  | 1st generation | 2nd generation | 3rd generation | 4th generation | Undefined |  |
| COC users without family history of VTE | | 68,443 | 417,602 | 140,967 | 176,911 | 8,386 |  |
|  | % | 96.29 | 96.83 | 96.55 | 96.82 | 95.01 |  |
|  |  |  |  |  |  |  |  |
| COC users with family history of VTE | | 2640 | 13651 | 5044 | 5816 | 440 |  |
|  | % | 3.71 | 3.17 | 3.45 | 3.18 | 4.99 |  |
| Total | Number of cases | 71,083 (8.5%) | 431,253 (51.4%) | 146,011 (17.4%) | 182,727 (21.8%) | 8,826  (1.1%) |  |
